# Grading Ductal Carcinoma In Situ (DCIS) of the Breast – What’s Wrong with It?

**DOI:** 10.1007/s12253-019-00760-8

**Published:** 2019-11-27

**Authors:** Gábor Cserni, Anita Sejben

**Affiliations:** 1 Department of Pathology, Bács-Kiskun County Teaching Hospital, Nyíri út 38, Kecskemét, H-6000 Hungary; 2 Department of Pathology, University of Szeged, Állomás u. 1, Szeged, H-6725 Hungary

**Keywords:** Breast cancer, Ductal carcinoma in situ, Grade

## Abstract

Ductal carcinoma in situ of the breast is a non-obligate precursor of invasive breast cancer, and at its lower risk end might not need treatment, a hypothesis tested in several currently running randomized clinical trials. This review describes the heterogeneity of grading ductal carcinoma in situ (DCIS). First it considers differences between low and high grade DCIS, and then it looks at several grading schemes and highlights how different these are, not only in the features considered for defining a given grade but also in their wording of a given variable seen in the grade in question. Rather than being fully comprehensive, the review aims to illustrate the inconsistencies. Reproducibility studies on grading mostly suggestive of moderate agreement on DCIS differentiation are also illustrated. The need for a well structured, more uniform and widely accepted language for grading DCIS is urged to avoid misunderstanding based misclassifications and improper treatment selection.

**Electronic supplementary material:**

The online version of this article (10.1007/s12253-019-00760-8) contains supplementary material, which is available to authorized users.

## Introduction - Something Personal, to Begin with…

The first author, among many other (about 50) participants, was asked to classify ductal carcinoma in situ (DCIS) along several features (10 “scored” variables) in a set of nearly 150 digital slides each representing a single case from a series of 353 DCIS with known outcome. This task was accepted voluntarily as the study framing this exercise has very ambitious and complex aims, which include sorting out some troubles of DCIS classification and possibly clarifying which cases could safely avoid the relatively uniform treatment of a heterogeneous disease summarized under the same name of DCIS. The study coordinated by Jelle Wesseling from the Netherlands Cancer Institute also looks at the possibility whether standard morphology is capable of sorting out, identifying these latter cases, which are likely to be overtreated with a “one size fits all” approach. So motivation, daily practice expertise (and beyond), a friendly relation with the coordinator of the study were all there to go ahead and score the cases. But then, at the first case, looking at the variables to score and the advice to follow everyone’s own routine made this author uncertain. By the end of the few hours spent on grading DCIS and classifying it otherwise, the sense of chaotic set of rules or chaos from the lack of rules and the feeling of being far away from perfectness resulted in frustration. This feeling initiated the writing of this review, which tries to give an impression on how far we are from a uniform approach to DCIS.

## DCIS as a Precursor of Invasive Carcinomas

Ductal carcinoma in situ (DCIS) is well accepted as a non-obligate precursor of invasive breast carcinomas. As invasive breast cancer is not a single disease, it is more than logical that its precursor cannot be one disease either. Invasive carcinomas are classified according to histological type, grade, biomarker expression, stage … etc. Similarly, DCIS is also classified along several features: e.g. pattern, grade, necrosis and biomarker expression.

It is currently held that low grade (well differentiated, grade 1) invasive breast carcinomas derive from low grade DCIS, whereas high grade DCIS is the precursor of high grade (poorly differentiated, grade 3) invasive carcinomas [1], and this – not surprisingly – suggests that LG DCIS and HG DCIS have different prognoses. For invasive carcinomas, the Nottingham grading scheme [2] has gained worldwide acceptance, and is generally recommended in guidelines [3–6]. This distinguishes between 3 histological grades with worsening prognosis. On the other hand, gene expression profiling studies have suggested a two-tiered prognostic separation, which lumps mostly histological grade 1 tumours and part of grade 2 tumours into a single group, the molecular (genomic) low grade category, and leaves the rest of histological grade 2 tumours and most grade 3 tumours for the molecular (genomic) high grade category [7]. This could mean that histological grade 2 tumours would reflect only our inability to classify these tumours as of better or worse prognosis on the basis of haematoxylin and eosin (HE) stained histological slides. Being a mixture of low and high genomic grade tumours, histological grade 2 carcinomas, as a group, obviously have intermediate prognosis between grade 1 and 3 tumours.

Similarly to invasive breast carcinoma, DCIS has also been found to be classifiable into two molecular grades with a split of intermediate histological grade DCIS cases into low and high molecular grades [8, 9]. In keeping with the above notion, while the low-grade pathway and high grade pathway seem generally accepted [1], there has been no proposition to state that intermediate grade DCIS gives ground to grade 2 invasive carcinomas, although this may be true in a number of cases.

As DCIS, by definition, does not give rise to metastases, its treatment could be local alone. By removing / abolishing a precursor lesion, one can prevent the disease which develops from it. On the other hand, as DCIS is a non-obligate precursor, removing lesions that will not progress to invasive carcinoma constitutes overtreatment. The low grade pathway mentioned before is not only associated with a better prognosis, but also with a slower rate of progression [10] and recurrence [11–13], and also a continuous rate of recurrence [9]. Some DCIS never progress to invasive carcinoma. By now, clinical studies have formulated the aim of proving that observation of low risk / low grade DCIS can have identical outcomes than (is not inferior to) surgical removal ± radiotherapy of these lesions [14–16]. The low risk DCIS trials (LORIS – LOw RISk dcis trial, LORD – LOw Risk Dcis trial and COMET - Comparison of Operative to Monitoring and Endocrine Therapy) have well stated inclusion (and exclusion) criteria [17]. Basically, they recruit screen detected patients presenting with microcalcification alone and diagnosed with either low grade (LORD) or low grade and low end of intermediate grade non-high grade (LORIS, COMET) DCIS on the basis of (large gauge) needle biopsies providing generous sampling. These patients are randomized between observation (i.e. active surveillance) and conventional surgery with or without radiotherapy with the possible addition of endocrine therapy in COMET [14–18].

But what do we or others mean by different grades of DCIS?

## Grading DCIS – A Babelian Mixture of Languages

**Fig. 1:**
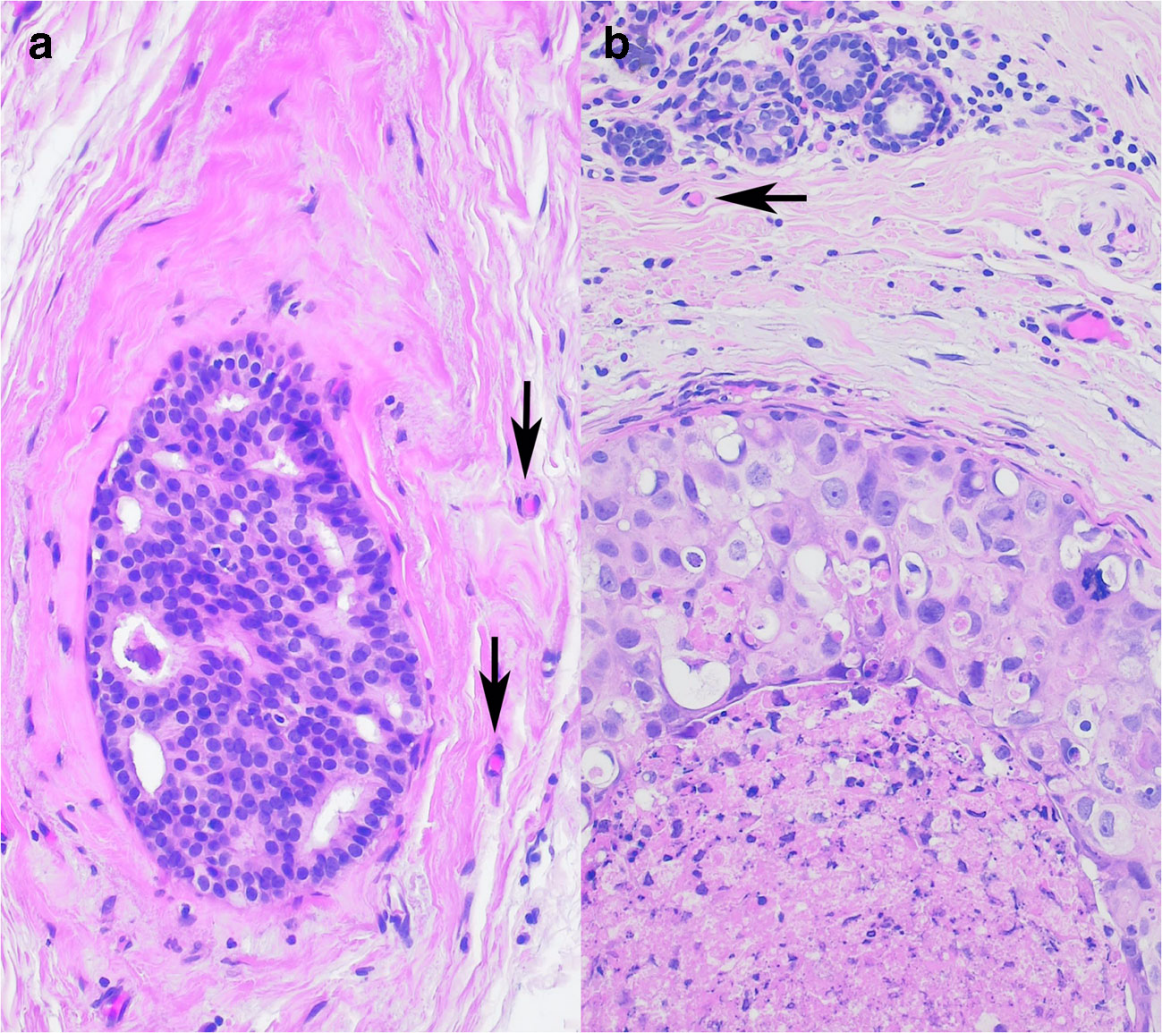
Examples of obvious low (**a**) and high (**b**) grade DCIS. Arrows point at single erythrocytes (RBC) in capillaries

**Fig. 2:**
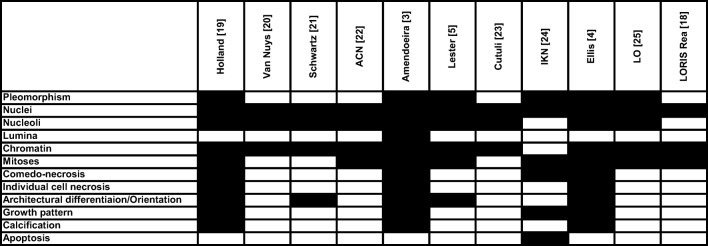
Features considered for grading DCIS according to different recommendations. ACN: Australian Cancer Network; IKN: Integraal Kankercentrum Nederland; LO: Leitlinienprogramm-Onkologie [3–5, 18–25]

**Fig. 3:**
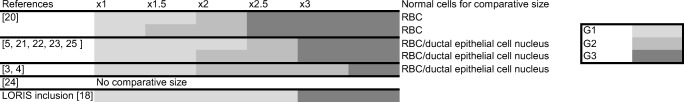
Comparative nuclear size described in some publications concerning the grading of DCIS. RBC: red blood cell / erythrocyte [3–5, 18, 20–25]

DCIS is traditionally graded as low, intermediate and high grade, and this is the recommendation made by the last edition of the European Guidelines [3]. There are cases, which are textbook examples of low grade (Fig. 1a) and high grade (Fig. 1b) DCIS, and probably even unexperienced pathologist would also reach a good concordance in classifying them according to grade. On the other hand, there are cases which give more pain for those trying to grade them. This must be partially related to the lack of uniformity in the guidelines for grading the disease. Figure 2 highlights the features considered while classifying DCIS into one of the grades according to some guidelines in use, and it shows that these features are not fully identical (The aim of Fig. 2 is to give a visual impression; Supplementary Table 1 summarizes the wording for the interested [3–5, 18–25]). Nuclear size criteria are included in many of these schemes, but as Fig. 3 highlights it, there is ground to interpret the cases differently [3–5, 18, 20–25].

A further source of confusion may stem from the terminology used. One of the first multinational European contemporary proposal for classification was the one by Holland et al., and this distinguished between well, intermediately and poorly differentiated DCIS [19]. As grade reflects differentiation in cancer, and invasive breast carcinomas of (histological) grade 1 are well differentiated, whereas those of (histological) grade 2 and 3 are moderately and poorly differentiated, respectively, it is tempting to equate the differentiation terminology with grade. But the generally accepted Nottingham grading system used for invasive breast carcinomas is a combined histological grade [2], in contrast to the nuclear grading system described by Black, which originally features a confusing (reverted) meaning of grades (grade 1 being the worst) [26]. The well-moderately/intermediately-poorly differentiated terminology for DCIS is based on cytonuclear and architectural differentiation [19], and therefore does not merely reflect the nuclear grade often used for grading DCIS. When one uses the term grade with a meaning corresponding to a set of complex nuclear and structural criteria (Fig. 2), reproducibility may suffer from the lack of weighting between these criteria: e.g. when the nucleus is not large enough to qualify for high grade, but structural features are those often seen in poorly differentiated DCIS, e.g. the lack of cellular orientation is conspicuous, there are many mitoses, necrosis is present.

To add to this confusion, the low risk (for recurrence, for invasive recurrence, for upgrading between needle biopsy and surgical specimen, for death after progression) of low risk DCIS is not only related to nuclear grade / differentiation, but also to other features like size / extent, margin involvement or proximity, age, proliferation. This has given rise to formulate prognostic indices, scores or multifactorial classifications [9, 20, 27–29] and molecular approaches.

## Reproducibility of Classifying DCIS

**Fig. 4:**
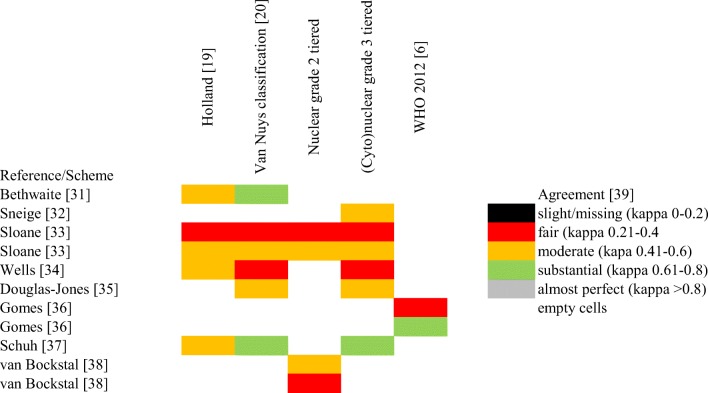
Different agreement levels reached in reproducibility studies on DCIS grade [6, 19, 20, 31–39]

As assessed by statistical means, the previously introduced and currently used recommendations for grading DCIS are far from being perfectly reproducible among observers. Several studies have looked at the consistency of grading or prognostically classifying DCIS, and generally found that these DCIS classifications were moderately reproducible, while a few others suggested worse or somewhat better agreement (Supplementary Table 2 and Fig. 4 representing it)[6, 19, 20, 30–39]. This degree of interobserver variability is also reflected by a recent analysis of nearly 5000 DCIS diagnosed during a period of 4 years in the Netherlands, and suggesting that the proportion of low grade DCIS varied between 6 and 24% by department. This is not so surprising as Fig. 4 reflects results gained with uniform classification criteria, whereas a questionnaire based assessment in the quoted Dutch study revealed a heterogeneity in the grading schemes used [40].

Although the values reflecting interobserver reproducibility for DCIS grading (Fig. 4) are not (much) worse than for many other features judged by microscopy (e.g. the grade of invasive breast carcinoma; the reporting of microinvasion in carcinoma in situ; the identification of lymphovascular invasion), they suggest that studies looking at the prognosis or prognostic markers of DCIS cannot ignore this degree of variability, and steps aiming at improving this are warranted.

As seen with other morphological features, a classification in a two-tiered system is always more consistent than one in a three-tiered system, simply by the fact that by chance identical categorization is greater. This is also exemplified by the study of Van Bockstal et al. [38], who found that reproducibility is improved when 2 categories are used instead of 3, but also highlighted that the distinction between high grade versus non-high grade was better (median Cohen’s kappa value: 0.53) than the distinction between low grade versus non-low grade (median Cohen’s kappa value: 0.39) (Fig. 4). This may be a worthwhile approach to improve reproducibility of the prognostic grouping of DCIS on morphological grounds, although as stated in the first section, the intermediate grade might reflect our less than perfect ability to distinguish between the good (low genomic grade, low risk) and the bad (high genomic grade, high risk).

## Further Inconsistencies

Beside the determination of differentiation, i.e. grading, there are other inconsistencies in recommendations concerning the classification of DCIS. For some reasons, the 1997 consensus conference recommendations have included a comedo pattern among the traditionally recognized non-special types of DCIS, therefore making 5 major pattern categories: comedo, solid, cribriform, micropapillary, papillary [21]. Others recognize “comedo” only as a type of necrosis, which can occur with the solid, but also with other patterns of DCIS [4]. For some, necrosis worth to be mentioned is only the central comedo type necrosis [4, 20], and punctate, focal necrosis needs no reporting, whereas the 1997 Philadelphia Consensus Conference recommendation, and national guidelines following these recommendations recognize non-comedo type (punctate) necrosis, too, and recommend reporting it separately [21, 23].

## Concluding Remarks

The fact that DCIS classification is not uniform and harmonious is well recognized, and the need to establish a uniform classification system was formulated before. This is even more stressed, when studies try to prove that low grade or non-high grade (low risk) DCIS may not require anything else than careful follow-up. It is imperative to delineate what people should mean by low grade / low risk DCIS, in order to have a uniform interpretation not only of specific cases, but also of study results. This may have an impact on patient treatment or omission of treatment.

It might perhaps be wise to introduce a new, previously unused and uncompromised terminology as well, as disciples of former schools may understand the same term differently, as suggested by our Figs. 2 and 3. It seems that some existing national or regional guidelines use the same sources to be followed for grading DCIS, but obviously there are differences even between European countries and within counties. The running trials aiming at clarifying the outcome of low-risk DCIS without treatment also use somewhat different inclusion criteria [17].

There is a need to sort out the heterogeneity in terminology and the development of a common language that would mean the same thing to all those who communicate on it in order to avoid misclassification and the ensuing possibility of mistreatment simply arising from misunderstanding.

## Electronic supplementary material

ESM 1 (DOCX 24.9 kb)

### Electronic supplementary material

ESM 2 (DOCX 15.5 kb)
