# Supplementary material for: Grading Ductal Carcinoma In Situ (DCIS) of the Breast – What’s Wrong with It?
Source: Pathol Oncol Res. 2019 Nov 27;26(2):665–71. doi: 10.1007/s12253-019-00760-8 (PMC7242244; doi:10.1007/s12253-019-00760-8)
Supplement: Supplementary file 1 — (DOCX 24.9 kb) [file 12253_2019_760_MOESM1_ESM.docx]

| **First author [Reference]** | **Aspects** | **Low grade DCIS** | **Intermediate grade DCIS** | **High grade DCIS** |
| --- | --- | --- | --- | --- |
| **Holland [19]*** | **Nuclei** | Monomorphic; Uniform size, regular outline and spacing | Pleomorphic +; Some variation in size, outline and spacing | Pleomorphic +++; Variation in size, irregular outline and spacing |
|  | **Nucleoli** | Insignificant | Evident | Prominent |
|  | **Chromatin** | Uniform, fine | Fine to coarse | Coarse, clumped |
|  | **Mitoses** | Rare | Occasionally present | Often present |
|  | **Architectural differentiation** | Marked | Present | Absent or minimal |
|  | **Central necrosis** | Absent or minimal | Variable | Usually present, often prominent |
|  | **Individual cell necrosis and autophagocytosis** | Absent | May be focally present | Usually present |
|  | **Growth pattern** | Clinging/ micropapillary/ cribriform or rarely solid | All patterns | Solid, clinging or pseudomicropapillary/cribriform |
|  | **Calcification** | Laminated, psammoma like or rarely amorphous | Amorphous or laminated | Amorphous |
| **Silverstein [20]** | **Nuclei diameter** | 1–1.5 RBC | 1.5–2 RBC | > 2 RBC |
|  | **Nucleoli** | No nucleoli | Infrequent | 1 or more |
|  | **Chromatin** | Uniform, fine | Fine to coarse | Vesicular |
| **Consensus conference committee [21]** | **Nuclei** | Monotonous (monomorphic); 1.5-2.0x size of a normal RBC or duct epithelial cell nucleus dimensions | Nuclei that are neither low grade or high grade | Markedly pleomorphic; >2.5x size of a normal RBC or duct epithelial cell nuclear dimensions |
|  | **Nucleoli** | Occasional |  | Prominent, often multiple |
|  | **Chromatin** | Diffuse, finely dispersed |  | Vesicular, irregular chromatin distribution |
|  | **Mitoses** | Occasional |  | May be conspicuous |
|  | **Orientation** | Polarization of constituent cells |  |  |
| **Amendoeira [3]** | **Pleomorphism** | Monomorphic, evenly spaced cells | Moderate | Pleomorphic, irregularly spaced and usually large exhibiting marked variation in size, irregular nuclear contours |
|  | **Cell size** | Small, 1-2x the size of a RBC | Typically larger than seen in low grade DCIS and are between 2 or 3 times the size of a RBC | Large and greater than 3x the size of a RBC |
|  | **Cellular composition** |  | Nuclear to cytoplasmic ratio is often high |  |
|  | **Nuclei** | Rounded, centrally placed |  |  |
|  | **Nucleoli** | Inconspicuous | 1 or 2 | Prominent |
|  | **Chromatin** |  |  | Coarse |
|  | **Lumina** | Well delineated, regular punched out lumina in cribriform type |  |  |
|  | **Mitoses** | Few |  | Usually frequent and abnormal forms may be seen |
|  | **Necrosis** | Rare individual cell necrosis |  | Comedo type central necrosis may be present |
|  | **Growth patterns** | Micropapillary or cribriform, less frequently solid | May be solid, cribriform or micropapillary | Often solid, may also exhibit micropapillary and cribriform patterns |
|  | **Cell orientation** | Polarization of cells covering the micropapillae or lining the intercellular lumina. | Cells usually exhibit some degree of polarization covering papillary processes or lining intercellular lumina | Rarely any polarization of cells covering the micropapillae or lining the intercellular spaces |
|  | **Calcification** | Usually punctuate (not a good discriminant) |  | Amorphous or clustered calcification may be present |
|  | **Comment** |  | Clear cell DCIS, apocrine DCIS often fall into this category | Large nuclear and nucleolar size does not preclude lower grade in apocrine lesions |
| **Australian Cancer Network [22]** | **Nuclei** | Size equivalent to 1.5–2 red blood cell diameters or normal duct epithelial nuclei | Size equivalent to 2–2.5 red blood cell diameters | Pleomorphic, vesicular, size > 2.5 red blood cell diameters |
|  | **Nucleoli** | Inconspicuous | Infrequent | One or more prominent |
|  | **Chromatin** | Diffuse | Coarse |  |
|  | **Mitoses** |  | Infrequent | Frequent mitotic figures commonly present |
|  | **Biological characteristics** |  |  | Absence of estrogen and progesterone receptor expression, aneuploidy, a high proliferative index, membrane reactivity for HER2, p53 nuclear expression and abnormal bcl2 expression |
| **Lester [5]** | **Nucleoli** | Only occasional | Intermediate | Prominent, often multiple |
|  | **Chromatin** | Usually diffuse, finely dispersed | Intermediate | Usually vesicular with irregular chromatin distribution |
|  | **Pleomorphism** | Monotonous | Intermediate | Markedly pleomorphic |
|  | **Nuclear size** | 1,5-2x the size of a normal RBC or a normal duct epithelial cell nucleus | Intermediate | >2.5x the size of a normal RBC or a normal duct epithelial cell nucleus |
|  | **Mitoses** | Only occasional | Intermediate | May be frequent |
|  | **Orientation** | Polarized toward luminal spaces | Intermediate | Usually not polarized toward the luminal space |
| **Cutuli [23]** | **Nuclei** | Monotonous (monomorphic); 1.5-2.0x size of a normal RBC or duct epithelial cell nucleus dimensions | Nuclei that are neither low grade or high grade | Pleomorphic; >2.5x size of a normal RBC or duct epithelial cell nuclear dimensions |
|  | **Nucleoli** | Few |  | Prominent, multiple |
|  | **Chromatin** | Finely dispersed |  | Heterogeneous |
|  | **Mitoses** | Few |  | Visible |
| **Integraal Kankercentrum Nederland [24]*** | **Cells** | Quite clearly cubic or cylindric cytoplasm | In between |  |
|  | **Nuclei** | Small, regular and round nuclei that do not overlap each other | In between | Enlarged, polymorphic |
|  | **Mitoses** | Little to no mitotic activity | In between | Evident mitotic activity |
|  | **Apoptosis** | Little to no apoptosis | In between | Evident apoptosis |
|  | **Necrosis** | Minimal | In between | Often central necrosis in largely solid epithelium |
|  | **Growth patterns** | Micropapillary or cribriform | In between |  |
| **Ellis [4]** | **Pleomorphism** | Monomorphic, evenly spaced cells | Moderate pleomorphism, lack of monotony of small cells | Pleomorphic, irregularly spaced and usually large, marked variation in size with irregular nuclear contours |
|  | **Nuclei** | Rounded, centrally placed; usually, but not invariably, small and are typically 1-2 times the size of a RBC | 2-3 RBC; N/C ratio often high | >3 RBC |
|  | **Nucleoli** | Inconspicuous | 1 or 2 | Prominent |
|  | **Chromatin** |  |  | Coarse |
|  | **Mitoses** | Few |  | Usually frequent and abnormal forms may be seen |
|  | **Necrosis** | Rare individual cell necrosis |  | Often comedo type necrosis |
|  | **Growth patterns** | Cribriform and micropapillary, less frequently solid | Solid, cribriform or micropapillary | Often solid; micropapillary and cribriform |
|  | **Calcification** |  |  | Frequent amorphous |
|  | **Orientation** | Polarization of cells covering the micropapillae or lining the intercellular lumina | Some degree of polarization covering papillary processes or lining intercellular lumina | Rarely any polarization of cells covering the micropapillae or lining the intercellular spaces |
|  | **Comment** |  | Clear cell DCIS, apocrine DCIS often fall into this category |  |
| **Leitlinienprogramm-Onkologie [25]** | **Nuclei** | Monotonous and isomorphic; 1,5-2 RBC or duct epithelial cell nucleus | Neither low grade nor high grade | Unequivocally pleomorphic; >2.5 RBC or duct epithelial cell nucleus |
|  | **Chromatin** | Diffuse, finely granular | Neither low grade nor high grade | Usually vesicular to irregular |
|  | **Nucleoli** | Only sometimes present | Neither low grade nor high grade | Prominent, often multiple |
|  | **Mitoses** | Rare | Neither low grade nor high grade | Sometimes striking |
