# Supplementary material for: Grading Ductal Carcinoma In Situ (DCIS) of the Breast – What’s Wrong with It?
Source: Pathol Oncol Res. 2019 Nov 27;26(2):665–71. doi: 10.1007/s12253-019-00760-8 (PMC7242244; doi:10.1007/s12253-019-00760-8)
Supplement: Supplementary file 2 — (DOCX 15.5 kb) [file 12253_2019_760_MOESM2_ESM.docx]

| **Author** | **Number of cases** | **Number of participants** | **Grading system** | **Statistics** | **Subjective reproducibility** |
| --- | --- | --- | --- | --- | --- |
| **Scott (30)** | 16 | 6 | Lagios Nuclear Grading System | high vs low grade category: agreement in 56% | "Consensus in a dichotomous classification was difficult." |
|  |  |  | Modified Lagios (with intermediate grade) | agreement in 94% of formerly discordantly classified cases |  |
| **Bethwaite (31)** | 25 | 11 | (Architectural classification) | kappa = 0.44 | moderate |
|  |  |  | Holland | kappa = 0.57 | moderate |
|  |  |  | Van Nuys | kappa = 0.66 | substantial |
| **Sneige (32)** | 125 | 6 | Modified Lagios (with intermediate grade) | kappa = 0.46 | "Moderate agreement." |
|  |  |  |  | grade 3 vs grade 2 kappa=0.48 |  |
|  |  |  |  | grade 1 vs grade 2 kappa=0.29 |  |
| **Sloane (33)** | 33 | 23 experts | Holland | kappa = 0.33 - 0.42 |  |
|  |  |  | nuclear grade (LG, IG, HG) | kappa = 0.28 - 0.41 |  |
|  |  |  | nuclear grade (HG vs non-HG) | kappa = 0.40 - 0.48 |  |
|  |  |  | Van Nuys | kappa = 0.33 - 0.48 |  |
|  |  |  | comedo necrotic vs non-necrotic | kappa = 0.27 - 0.39 |  |
| **Wells (34)** | 40 | 7 nonexpert pathologists | Holland | nonexpert: kappa = 0.46; expert: kappa = 0.53 | "Diagnostic reproducibility was highest using Holland." |
|  |  | 3 expert pathologists | Van Nuys | nonexpert: kappa = 0.26; expert: kappa = 0.29 | Intermediate grade: Holland and Lagios |
|  |  |  | Modified Lagios (with intermediate grade) | nonexpert: kappa = 0.26; expert: kappa = 0.29 | High grade: Van Nuys |
| **Douglas-Jones (35)** | 60 | 19 | cytonuclear grading | unweighted kappa = 0.36; weighted kappa = 0.47 | "Only moderate agreement." |
|  |  |  | Van Nuys | unweighted kappa = 0.40; weighted kappa = 0.48 |  |
| **Gomes (36)** | 610 | ?? | WHO classification [4th edition, 2012] |  | "The best diagnostic agreements were observed for high-grade DCIS, ALH and LCIS.**"** |
|  |  |  | low-grade DCIS | kappa = 0.47 |  |
|  |  |  | intermediate-grade DCIS | kappa = 0.45 |  |
|  |  |  | high-grade DCIS | kappa = 0.68 |  |
| **Schuh (37)** | 43 | 3 | Holland | intraobserver kappa = 0.57 | "Overall, diagnostic reproducibility was similar for all histologic grading classification systems." |
|  |  |  | Van Nuys | intraobserver kappa = 0.67 |  |
|  |  |  | Black Modified Nuclear Grade | intraobserver kappa = 0.67 |  |
| **Bockstal (38)** | 153 | 13 | CAP |  | "In this study, dichotomization of nuclear grade as non-high versus high was more reproducible and thus more robust than dichotomization as low versus non-low.**"** |
|  |  |  | nuclear grade | Krippendorff's alpha=0,5629 |  |
|  |  |  | high grade vs non-high grade | median pairwise kappa (range) = 0.53 (0.28-0.67) |  |
|  |  |  | low-grade vs non-low grade | median pairwise kappa (range) = 0.39 (0.04-0.64) |  |
